# Supplementary material for: The interactome of intact mitochondria by cross-linking mass spectrometry provides evidence for coexisting respiratory supercomplexes
Source: Mol Cell Proteomics. 2017 Dec 8;17(2):216–32. doi: 10.1074/mcp.RA117.000470 (PMC5795388; doi:10.1074/mcp.RA117.000470)
Supplement: Supplemental Data [file supp_RA117.000470_133922_0_supp_23978_fzffwf.zip › spectra_annotation/mito_spectra_annotation/339-1-6-1-7-1.pdf]

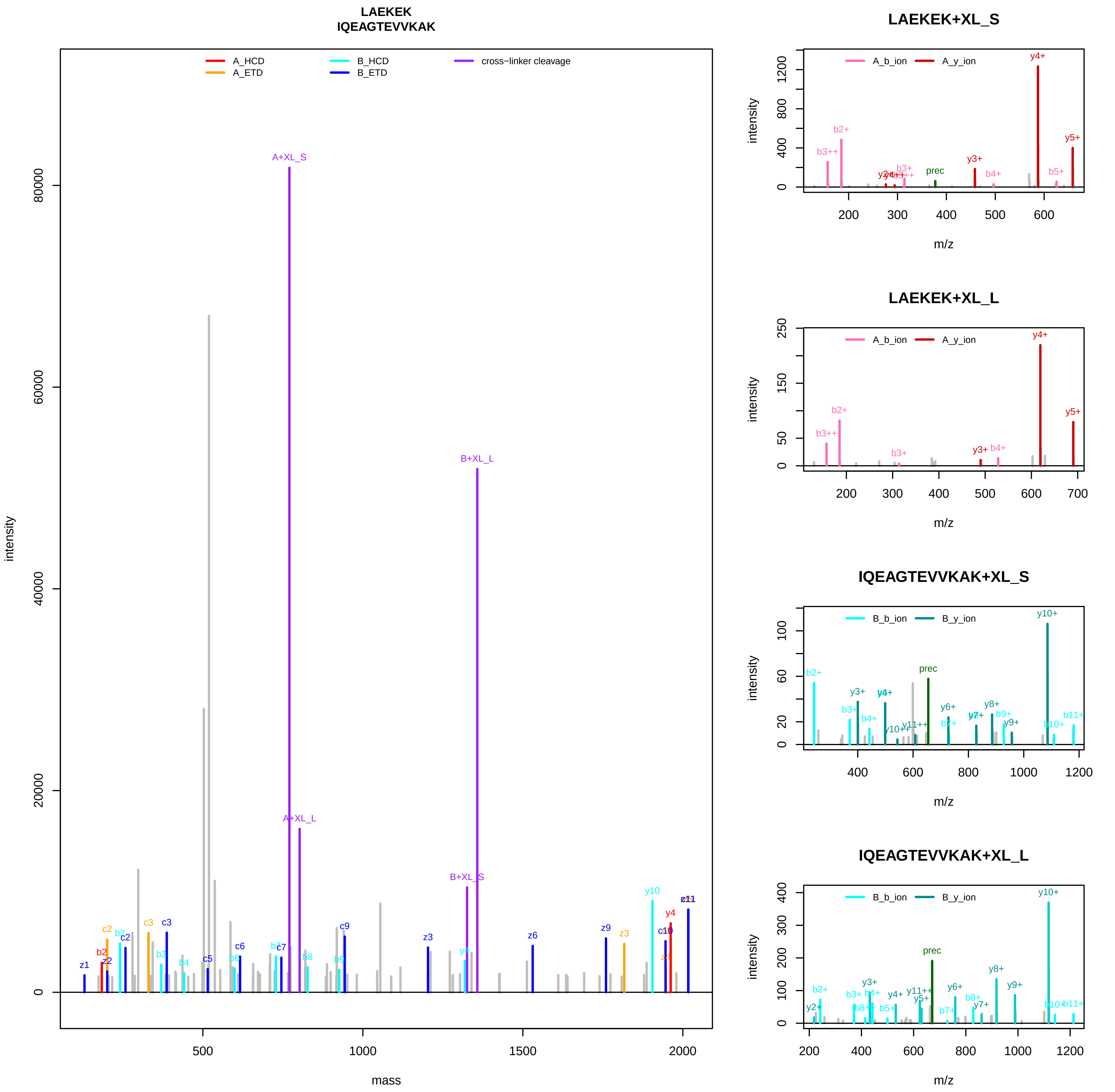
LAEKEK
IQEAGTEVVKAK
A_HCD
A_ETD
B_HCD
B_ETD
cross−linker cleavage
A+XL_S
B+XL_L
A+XL_L
B+XL_S
y10
y4
c11
z11
c10
z4
z3
z9
z6
z3
c9
b9
y4
b8
c7
b7
b6
c6
c5
b4
b3
c3
c2
b2
z2
z1
c3
c2
b2
intensity
mass
0
20000
40000
60000
80000
500
1000
1500
2000
LAEKEK+XL_S
A_b_ion
A_y_ion
y4+
b2+
b3++
y5+
y3+
b4+
b3+
b5+
prec
intensity
m/z
0
400
800
1200
200
300
400
500
600
LAEKEK+XL_L
A_b_ion
A_y_ion
y4+
b2+
b3++
y5+
y3+
b4+
b3+
intensity
m/z
0
50
150
250
200
300
400
500
600
700
IQEAGTEVVKAK+XL_S
B_b_ion
B_y_ion
y10+
b2+
prec
y3+
y4+
b3+
b4+
y6+
y7+
y8+
b9+
y9+
b7+
b10+
b11+
y11++
y10++
intensity
m/z
0
20
60
100
400
600
800
1000
1200
IQEAGTEVVKAK+XL_L
B_b_ion
B_y_ion
y10+
prec
y8+
y9+
y3+
y4+
y11++
b2+
b3+
b4+
y6+
y5+
b8+
b5+
y2+
b7+
y7+
b10+
b11+
intensity
m/z
0
100
200
300
400
200
400
600
800
1000
1200
